# Women’s experiences of dealing with fertility and side effects in contraceptive decision making: a qualitative study based on women’s blog posts

**DOI:** 10.1186/s12978-023-01642-8

**Published:** 2023-06-29

**Authors:** Lydia Johansson, Julia Vesström, Siw Alehagen, Helena Kilander

**Affiliations:** 1 Division of Nursing Sciences and Reproductive Health, Department of Health, Medicine and Caring Sciences, Linköping University, Linköping, Sweden; 2 Jönköping Academy for Improvement of Health and Welfare, School of Health and Welfare, Jönköping University, P.O. Box 1026, 551 11 Jönköping, Sweden; 3 Department of Women’s and Children’s Health, Karolinska Institutet, and the WHO Collaborating Centre, Karolinska University Hospital, Stockholm, Sweden

**Keywords:** Birth control, Blogs, Contraceptive counselling, Fertility awareness, Thematic analysis

## Abstract

**Background:**

Worldwide, there is limited knowledge regarding women’s views of future fertility in relation to contraceptive use. Few studies include material where women share their experiences at peer-written public domain websites, in spite of a larger portion of women discontinuing use of contraceptives. The objective of this study was to explore women’s experiences of contraceptive methods based on data gathered from individual blog posts.

**Methods:**

Explorative qualitative study including 123 individual blog posts as the data source analysed with inductive thematic analysis.

**Results:**

Two themes were identified. Theme 1, ‘Seeking control over reproduction and optimise fertility*’* including the sub-themes; Having the possibility to decide if, and when, to become pregnant, The value of effective contraceptive methods and the impact of women’s sexuality, A wish to understand the body’s normal fertility function and Limited knowledge—sharing information about the menstrual cycle during counselling and Theme 2, ‘*Making the complex decision on their own’* including the sub- themes; Limited or subpar guidance in counselling and need for information from social media, Relational and environmental factors influencing contraceptive decision making and Considering beneficial effects and fears of adverse health effects when using hormonal contraceptive methods.

**Conclusions:**

During counselling, women desired an extended dialogue regarding effectiveness, health effects of different methods and an increased understanding of their menstrual cycle. Insufficient understanding of contraceptive methods can lead to use of methods not providing the expected level of protection. Hormonal contraceptives, especially Long-acting reversible contraception (LARC) were believed to inhibit fertility long after ending treatment.

## Background

Women’s needs and choices of contraception change over time and contraception seems to be a complex issue in women’s fertile lives [1]. A previous review including studies from continents such as Europe, America, Asia and Africa show low to moderate fertility awareness among both women and men in their 20 s and 30 s, reflecting needs of increased understanding [2]. Women who postpone childbearing also report needs for knowledge on how to optimise future fertility and how to avoid unintended pregnancy [3]. There is still limited understanding regarding how women reason and deal with fertility in relation to choose and use of contraceptive methods.

According to studies in both Europe, Africa, Asia and Latin-America, discontinuation of hormonal contraceptives during the first year of use is common [4–8]. Women describe, discontinuation due to experiences of health concerns [4] such as irregular bleeding patterns [4–6], weight gain, headache [4], and negative effects on mood and sexual desire [7, 8]. Among women who choose LARC, discontinuation within two years also seems common due to experiences of health concerns [9].

Worldwide, women’s choices of contraceptive method seem to be a sensitive decision that is influenced by many factors [10, 11] such as contraceptive characteristics [10], women’s reproductive history and previous experiences of using contraception. Furthermore, social aspects and norms such as partner’s support [11, 12], family members expectations, [11], reported experiences among friends [11, 12] and in social media [13, 14] affect women’s choice and use.

Social media plays a central role in contraceptive choice, where women share anecdotal experiences of contraception through for example, videos [13] and tweets [14], in addition to the traditional social networks of family and friends [13, 14]. The majority of research on women’s experiences of contraception is based on questionnaires, interviews or focus groups [1, 4, 7]. Exploring blog posts in which women share experiences, offer new information not revealed by other sources, and access to women’s views and conversations between each other in online communities without the impact of pre-defined research questions [15].

To our knowledge, despite a greater portion of women choosing to discontinue use of contraceptives resulting in an increased risk of unintended pregnancy, only a few studies use material regarding women’s views in contraceptives from peer-written public domain websites [13, 14].

Studying blogs gives a unique access to blogger’s experiences without influence of pre-defined research questions. This may give a new understanding of women’s experiences of contraception which is needed in developing contraceptives services, so they are designed to better fulfil women’s needs. Hence, the aim of this study was to explore women’s experiences of contraceptive methods based on data gathered from individual blog posts.

## Methods

### Design

The design was an explorative qualitative study using blogs as the data source.

### Setting

This study was based on data from Swedish blogs. In Sweden, midwives and physicians are providers involved in contraceptive services. Midwives prescribe and administer the majority of contraceptive methods including LARC [16].

### Data collection

Data from the web can present new and valuable information of a phenomenon. Exploring blog posts in which women share experiences is describes as unfiltered self-narratives. Blog posts can offer new information not revealed by other sources, and access to women’s views without the impact of pre-defined research questions [15].

The blogs were identified by using the search engine Google. Purposive sampling was used to capture unfiltered self-narratives written by women with lived experiences of choosing and using contraceptive methods. The inclusion criteria were blogs written in Swedish documenting women’s own experiences of contraceptives. Exclusion criteria were blogs with restricted access e.g. protected by passwords or requiring account login, blog posts older than 5 years and commercial blog post with paid sponsorships. Photos, videos and comments were excluded.

A sample search was performed during July 2019 and it indicated that there were many blogs which responded to the aim of the present study. Data collection was performed from August 26th to September 10th 2019, by the authors LJ and JV.

Several combinations of search terms such as “contraceptives”, “contraceptive pill”, “condom” and “pessary”, were used and combined with Boolean operators ‘AND’ and ‘OR’. In total, 19 combinations of search terms were used and four of these resulted in the majority of the blog posts that were included in the analysis. These combinations were: “started with contraceptives, blog”, “ended with contraceptives, blog”, “hormonal contraceptives, blog” and “contraceptives*,* contraceptive implants*,* intrauterine devices*,* blog*”.* None of the blogs were solely about experiences of contraceptives and therefore other types of content were manually excluded. Finally, 58 pages of data were used in the analysis which included 140 blog posts written by 123 women. The sample size of 123 women was due to the amount of data in each blogpost. In total, 112 presented themselves by name and 39 stated their age, which ranged between 16 and 48 years of age (median age 25 years). Some women mentioned their place of residence representing a geographical spread from northern to southern Sweden.

### Data analysis

A thematic analysis according to Braun and Clarke was chosen and carried out in six steps [17]. Thematic analysis is a suitable method for an inductive approach aimed to identify, analyse and report pattern in materials. The analysis is a recursive process where the movement is back and forth through all the steps. The first and second authors (LJ, JV) were responsible for the analysis. To strengthen the trustworthiness of the study, a pilot analysis was performed and discussed among all four authors. In the first step, the authors read the data to familiarise themselves with the content and to identify thoughts and patterns in the data. Data which correspond to the aim, was selected and this resulted in 38,996 words and 58 pages. In the second step, the data extracts were labelled, and preliminary codes were created. In the third step, preliminary codes were then organised into 23 preliminary themes and were discussed and grouped together. In the fourth step these 23 preliminary themes generated seven sub-themes. In the fifth step these seven sub-themes were grouped into two main themes, which constitute the findings (Table 1). The main themes were discussed among all four authors until consensus was reached. Finally, the six step is about the final analysis producing the report,

**Table 1:** Sub-themes and main themes

Sub-themes	Main themes
Having the possibility to decide if, and when, to become pregnant	*Seeking control over reproduction and optimise fertility*
The value of effective contraceptive methods and the impact of women’s sexuality	
A wish to understand the body’s normal fertility function	
Limited knowledge—sharing information about the menstrual cycle during counselling	
Limited or subpar guidance in counselling and need for information from social media	*Making the complex decision on their own*
Relational and environmental factors influencing contraceptive decision making	
Considering beneficial effects and fears of adverse health effects when using hormonal contraceptive methods	

The 15-point “Checklist of criteria for good thematic analysis” (Braun & Clarke, 2006, page 96) [17] was used throughout the analysis process to ensure that a good and reliable thematic analysis was provided. For example, all data was given equal attention during the coding process and themes were checked against the original data set. The data was analysed and interpreted in order to find underlying meaning contained within it. In qualitative methods it is crucial to balance the subjective influences of individual researchers [18], therefore all authors were involved in the analysis. The data collection and the analysis are clearly presented to strengthen trustworthiness of the research methods.

### Ethical considerations

The internet has been used as a site for research for some decades. Ethical challenges, such as participants’ anonymity and informed consent have been discussed and recommendations have been given. For example, by the Association of Internet Researchers (2019) [19]. Text on the internet, such as blogs, can be seen as public data and it may be implicitly assumed that bloggers have given their consent for these to be used [20]. The advisory board for studies conducted within the frame of university education granted oral approval for the study according to standard procedure (date of approval: 21 December 2018).

To ensure anonymity we do not specify in detail the terms we used in data collection, and only short parts of the blog posts were used as quotes in which some words have been rephrased.

## Results

The analysis resulted in two main themes: seeking control over reproduction and optimise fertility and making the complex decision on their own. Quotes are used in the results to illustrate the connection between the themes and the statements from blog-posts.

### Seeking control over reproduction and optimise fertility

Women described efforts to control their reproduction throughout their fertile lives. Reproductive control was described as a sense of being able to decide if, or when, they wish to become pregnant which in turn affected their sexuality. For a good sense of reproductive control, women reported wanting to use an effective method. This was described as improving their sexuality by facilitating their ability to relax and enjoy sexual intercourse. The fear of forgetfulness when using oral contraceptives meant that some women had difficulties in experiencing reproductive control. Several of them stated that they found LARC to be carefree by being a ‘forgettable’ method of contraception, which contributed to increased reproductive control.

*“So... I’ll insert a hormonal IUD that hopefully will remain there for five years, it would be a dream to not have to think about protection for five years—totally insane”*

Woman 26

Reproductive control was also described as being able to decide the duration between pregnancies and at the same time optimise fertility. Use of hormonal contraceptives, especially LARC, was believed to inhibit their fertility long after discontinued use, which concerned women who wanted to conceive in the near future. For example, if they wanted a shorter time span between pregnancies than three years, many women found LARC unsuitable and therefore refrained from its use despite the need of an effective contraceptive method. The importance of good reproductive control was also described in relation to certain phases of life, when women value the effectiveness of hormonal methods highly enough that they tolerate perceived side effects that these methods may cause.

Women described a lack of knowledge regarding the effectiveness of contraceptive methods and possible effects on fertility after discontinued use. Some stated feelings of increased reproductive control when they combined several non-hormonal contraceptive methods, such as pessary and another barrier method or the Billing’s (ovulation) method, hoping that this would lead to increased effectiveness.

*“I think we will try to become pregnant after the summer, but it’s not planned yet…I need a contraceptive method but at the same time I want to be able to conceive quickly after discontinuing it… when I used the implant it took a while before my period returned and became regular.”*

Woman 27

The women also expressed a need for increased awareness of how fertility works to be able to optimise fertility. This was considered important in order to experience reproductive control and an understanding of how to avoid unintended pregnancies. Fertility awareness was expressed as contributing to an increased feeling of control and security and was described by women as knowledge about the ‘fertile window’, phases of the menstrual cycle and changes in the body. The ability to identify the fertile window based on how cervical secretion changes during the cycle was described as important. They stated that knowledge-sharing and dialogue regarding the fertile window was often lacking during contraceptive counselling with the provider, which was experienced as a weakness in the counselling.

*“Personally, I’ve felt very ill from hormones and I’ve felt that there was very little conversation about the menstrual cycle, how the body works and other contraceptive options. Many times women’s negative experiences of hormones are simply brushed off. For me these subjects have been vitally important and therefore I have wanted to speak openly about it.”*

Woman 37

*“It would have been nice to use a method without hormones, but I probably won’t dare to try using one without hormones until I feel that it doesn’t matter that much if I get pregnant or not.”*

Woman 10

Lack of fertility awareness is described leading to anxiety about risking unintended pregnancies during the fertile life. Women described how they gained an understanding of their menstrual cycle by discontinuing use of hormonal contraceptives and switching to NFP. For example, the use of mobile phone applications for NFP contributed to an increased understanding of the fertile window leading to increased awareness.

Inadequate fertility awareness was described as a barrier for some women to start using NFP. Women using NFP successfully long term described that time was needed to learn how the method works, motivation to strictly follow the cycle pattern daily and to live with regular routines. Otherwise, there was a risk of discontinuation and a lack of reproductive control.

*“I can only become pregnant a few days per month… I started using NFP and understood my fertility and menstrual cycle. Imagine all the times in my life I’ve been worried sick about being pregnant because I had unprotected sex. Nobody had ever told me …I thought I could become pregnant any day of the month”*

Women 102

### Making the complex decision on their own

Women’s choice of contraceptive method was described as a complex decision they had to make on their own. Making the decision included the process of identifying a suitable contraceptive method among a variety of options that suit their needs in each phase throughout their fertile life. The complexity involved both a fear of adverse health effects when using contraceptive methods and limited knowledge regarding adverse or beneficial health effects. In addition, women described that lack of knowledge sharing and guidance in counselling sessions contributed to the complexity.

Having a variety of options was appreciated, but it also contributed to making the decision more complex. Trying several different methods before finding a suitable one, not having a natural menstrual cycle or being without a period during the use of a hormonal contraceptive, was described as exposing their bodies to medical experiments.

*“I’m so tired of hormones... totally insane to experiment with women’s period which doesn’t even come due to ovulation. Menstruation is a great sign of health! There are so many side effect (with birth control pills)... so many women suffer... it feels so out of date!”*

Women 71

Several factors were considered in the complex decision-making process, such as environmental aspects of the chosen method and the need for sharing the responsibility using contraception with a partner. Women described for example, concerns about synthetic hormones being released into the water cycle and as a result affecting wildlife negatively.

*“It will soon probably become a big environmental problem considering all the hormones that people are peeing out into our water”*

Women 102

They also took into consideration the risk of their partner feeling an IUD during intercourse. Women stated that limited support and lack of responsibility or involvement from the partner was burdensome. They felt forced by the expectations from providers and partners to take responsibility and make the decision on their own. Women described that they were dissatisfied with having to risk their well-being or to deal with side effects for the benefit of their joint sexuality.

*“Unfortunately, the options are so few which forces most young girls to begin with hormonal contraceptives. We must really hope they come up with a better option, maybe even a contraceptive for men. Why should it really be up to women to prevent pregnancy? Isn’t it just as much men’s task to make sure they protect themselves?”*

Women 50

By having to consider the need for effective contraception alongside possible beneficial health effects as well as a fear, or risk, of adverse health effects such as cancer or thrombosis was described to make the decision more complex. Women described that they used hormonal methods to achieve beneficial health effects, for example, to decrease heavy menstrual bleeding. At the same time, they battled fears of adverse health impacts, which caused them worry and confusion. Some women avoided choosing hormonal methods due to fear of unnatural hormonal imbalances. For example, they stated that supplying their bodies with synthetic substances was unnatural as it affected the menstrual cycle, especially when leading to the absence of menstruation.

*“Today I have a contraceptive implant that has given me […] two years of constant periods. Two years (!!!). But we women are expected to put up with it, we are expected to carry the responsibility.”*

Women 25

An expressed scepticism and fear of carrying foreign objects in the body or supplying it with synthetic hormones or what were believed to be toxic levels of copper, complicated the choice of contraception. The application of LARC also made the decision more complex due to concerns of pain during insertion or withdrawal. This deterred some women from choosing LARC, especially if they planned to use the method for a shorter time. Involved support from the provider, both during the decision-making process and at the time of insertion of an IUD, was of great importance and described as leading to an increased sense of security and improved the experience of insertion.

*It was when I switched to a new IUD that … many more problems started to occur. Pain in the hips, joint pains, especially when I worked out… I can also tick off the majority of the symptoms what the other 2500 members of the Copper group [on facebook] stated. Some of them are fatigue, tense muscle, low iron levels, trembling in the chest, hair loss and breasts so tender that they couldn’t be touched.”*

Women 5

Women expressed limited knowledge about contraceptive methods, which made it difficult to carry out an informed decision. They stated insufficient information and guidance during counselling and described how they felt that the providers did not actively listen. Their experiences of providers ignoring or belittling women’s self-perceived side effects or limiting and excluding information about possible negative side effects, complicated the decisions.

*“She (the provider) emphasised how big the risk is of becoming pregnant when using condoms (eh, since when?). I really had to fight to even get my voice heard. In the end I said if I become pregnant it happens, I don’t want any pills”. There was no other way. […] She (the provider) just couldn’t accept that I wanted to stop using the pills.”*

Women 8

Some women described that it could be easier to tolerate side effects if they received information about them during the counselling sessions. Women frequently sought information via social media, podcasts and internet forums instead.

*“..I Googled around and copper poisoning came up. I ended up on a blog about poisoning by copper IUDs and so much coincided with me!”*

Women 71

## Discussion

The theme seeking control over reproduction and optimise fertility highlights the value of having an effective contraceptive method, as it reduces women’s concern regarding unintended pregnancy and helps them to decide when to conceive. This finding in a Swedish context is strengthened by previous studies from other continents [10, 11] showing that a large proportion of women consider the effectiveness of the contraceptive method to be the most important factor in their choice of contraception [10, 11, 21]. Our study also illustrates women’s fears of using hormonal contraceptives, especially LARC, due to the belief of it inhibiting fertility long after discontinuing use. These findings emphasise the importance of communicating both the effectiveness of different contraceptive methods and also how quickly fertility returns after ending treatment, in a clear and comprehensible manner during counselling. To have reproductive control also influences women’s sexual experiences, an aspect which should be considered in contraceptive counselling.

Additionally, the theme also points out a knowledge gap and yearning for greater fertility understanding, which also has been reported by women in many different continents such as Europe, America, Asia and Africa [2, 22]. Some women in our study changed from hormonal methods to NFP because they wished to gain a better understanding of their bodies and control over their fertility. This phenomenon could be related to being influenced by other women in a Swedish social media community as well as a higher age among first time mothers in Sweden (30 years) [23]. However, the phenomena illustrates a need of dialogue, strengthening women’s fertility awareness no matter country of origin. The topic of fertility in relation to contraceptive methods could be improved during contraceptive counselling; especially as many mobile fertility applications are designed to neither avoid pregnancies nor use evidence-based methods. Instead, they use their own algorithms that can be difficult to assess as they have often not been reviewed in peer-review studies [24]. Hence, counselling could be developed by integrating fertility awareness, contraceptives effects on fertility after ending treatment and illustrations of the effectiveness of different contraceptive methods into the session.

Overall, the theme making the complex decision on their own, stresses a need for designing contraceptive services by including the components outreach and building trust, quality, access and follow-up support [25]. This is likely relevant when organising contraceptive services despite cultural context, since choice of contraceptive methods is a sensitive decision for many women worldwide [1, 10, 11]. Need of support and building trust should be considered when developing systems aimed to facilitate dialogues between people and health care providers. These needs are for example illustrated in women’s general fear and scepticism towards use of hormonal contraception and copper IUDs causing predominantly adverse reactions, as well as fear of cancer due to hormonal contraceptives. However, neither of these fears are supported by current research [26] and could be met in dialogues with providers in online services where women share their worries according to our findings.

Furthermore, needs of developing quality in counselling and follow-support [25] for side effect management and to facilitate method switching, was highlighted among women who stated going through several methods before finding a suitable one, and how it was exposing their bodies to medical experiments. A better quality and follow up support could likely prevent unnecessary worry or discontinuation of methods, especially as women in our study described that they could more easily tolerate side effects if they received information about them during the counselling session. Similar findings are previously reported [27], which showed how women who received information about side effects were more likely to continue with the chosen method. At the same time, needs of importance, such as individualised and non-coercive counselling [25] was illustrated in this theme as women doubted whether providers were actively listening during contraceptive counselling. This finding is consistent with previous studies showing that women prefer a person-centred approach in which active listening and support is a key component [27, 28]. Our study also indicates the importance of quality in providers; guidance and support when choosing an IUD, both during counselling and at the time of insertion, also reported elsewhere [29].

As a qualitative explorative study there are methodological limitations. Using collected data from public blog posts, where identity and socio-demographic parameters cannot be confirmed have limitations due to the authors of the blogs not being able to be contacted to validate the research findings [18].On the other hand, as blog posts can be published without the person having to state their identity, the blog authors can share feelings and experiences in a more open way than in, for example, personal interviews [20]. In Sweden, people between the ages of 16–55 years most commonly write and read blogs and more than 50 percent of this age group read other people’s blogs [30]. Although only 32 percent of the participants in our study provided their age, their ages were between 19 and 48 years, which is representative of the women who write and read blogs [30].

Prior to this study, studies on blog posts focusing on women’s experiences of contraceptive methods are sparse. Our findings represent 123 individual women’s self-describes experiences, whose geographical spread extends across Sweden, which may contribute to our findings being transferable to women’s views of contraction on social media [18].

A disadvantage of blog studies may be that only a selection or subgroup of the target group write blogs. Women struggling in finding contraception that works for them may be more likely to write blog posts on contraception. However, unfiltered experiences from blog posts are difficult to find on other studies and are of importance when seeking to improve and design contraceptive services for women who struggle and probably have higher risk of unintended pregnancy.

Future research should focus on how to co-design systems for contraceptive services together with women and men with different cultural background, by including the components outreach, building trust, quality, access and follow-up support.

## Conclusions

Contraceptive methods have a profound effect on women’s fertile lives. When striving for reproduction and fertility control, the effectiveness of the contraceptive method was highly valued. In contrast, some women believed that hormonal methods and LARC inhibit fertility long after the end of treatment. There appears to be a lack of knowledge regarding fertility awareness and women express an unmet need to have a dialogue about these issues during contraceptive counselling. Women’s choice of contraceptive method is based on a complex decision-making process on their own. They described a fear of using synthetic hormones which partially can be based on lack of knowledge and a concern regarding side effects. This can lead to women using contraceptive methods that do not provide the protection against unintended pregnancy that they are seeking. A solution could be to better address women’s fears of hormonal contraceptives during counselling and develop follow-up support online services in order to clarify misconceptions, prevent unnecessary discontinuation and facilitate method switching**.**

## Data Availability

The dataset generated and/or analysed during the current study is not publicly available due to restrictions from the advisory board for studies conducted within the frame of university education but can be made available to qualified researchers upon request, after approval from the advisory board. HK should be contacted to request the data.

## Abbreviations

NFP: Natural Family Planning
LARC: Long-acting reversible contraception
SARC: Short-acting reversible contraception

## References

[1] Downey MM, Arteaga S, Villasenor E, Gomez AM (2017). More than a destination: contraceptive decision making as a journey. Womens Health Issues.

[2] Pedro J, Brando T, Schmidt L, Costa ME, Martins MV (2018). What do people know about fertility? A systematic review on fertility awareness and its associated factors. Ups J Med Sci.

[3] Skogsdal Y, Karlsson JA, Cao Y, Fadl HE, Tyden TA (2018). Contraceptive use and reproductive intentions among women requesting contraceptive counseling. Acta Obstet Gynecol Scand.

[4] Fruzzetti F, Perini D, Fornaciari L, Russo M, Bucci F, Gadducci A (2016). Discontinuation of modern hormonal contraceptives: an Italian survey. Eur J Contracept Reprod Health Care.

[5] Hellstrom A, Gemzell Danielsson K, Kopp Kallner H (2019). Trends in use and attitudes towards contraception in Sweden: results of a nationwide survey. Eur J Contracept Reprod Health Care.

[6] Cohen R, Sheeder J, Teal SB (2019). Predictors of discontinuation of long-acting reversible contraception before 30 months of use by adolescents and young women. J Adolesc Health.

[7] Malmborg A, Brynte L, Falk G, Brynhildsen J, Hammar M, Berterö C (2020). Sexual function changes attributed to hormonal contraception use—a qualitative study of women experiencing negative effects. Eur J Contracept Reprod Health Care.

[8] Wood SN, Karp C, Zimmerman L (2020). Women’s sexual experiences as a side effect of contraception in low- and middle-income countries: evidence from a systematic scoping review. Sex Reprod Health Matters.

[9] Saloranta TH, Gyllenberg FK, But A, Gissler M, Laine MK, Heikinheimo O (2020). Free-of-charge long-acting reversible contraception: two-year discontinuation, its risk factors, and reasons. Am J Obstet Gynecol.

[10] Wyatt KD, Anderson RT, Creedon D, Montori VM, Bachman J, Erwin P (2014). Women’s values in contraceptive choice: a systematic review of relevant attributes included in decision aids. BMC Womens Health.

[11] D’Souza P, Bailey JV, Stephenson J (2022). Sandy Oliver Factors influencing contraception choice and use globally: a synthesis of systematic reviews. Eur J Contracept Reprod Health Care.

[12] Brown BP, Chor J, Hebert LE, Webb ME, Whitaker AK (2019). Shared negative experiences of long-acting reversible contraception and their influence on contraceptive decision-making: a multi-methods study. Contraception.

[13] Nguyen BT, Allen AJ (2018). Social media and the intrauterine device: a YouTube content analysis. BMJ Sex Reprod Health.

[14] Merz AA, Gutiérrez-Sacristán A, Bartz D, Williams NE, Ojo A, Schaefer KM (2020). Population attitudes toward contraceptive methods over time on a social media platform. Am J Obstet Gynecol.

[15] Hookway N (2008). Entering the blogosphere: some strategies for using blogs in social research. Qual Res.

[16] Swedish Medical Products Agency, 2014. Antikonception—behandlingsrekommendation. 2014;25(2):14 28. https://www.lakemedelsverket.se/sv/behandling-och-orskrivning/behandlingsrekommendationer/sok-behandlingsrekommendationer/preventivmetoder-for-antikonception--behandlingsrekommendation. Accessed 3 Feb 2023.

[17] Braun V, Clarke V (2006). Using thematic analysis in psychology. Qual Res Psychol.

[18] Flick U (2018). An introduction to qualitative research.

[19] Associations of internet researchers. Internet research Ethical Guidelines 3.0 2019 https://aoir.org/reports/ethics3.pdf. Accessed 3 Feb 2023.

[20] Eastham LA (2011). Research using blogs for data: public documents or private musings?. Res Nurs Health.

[21] Marshall C, Guendelman S, Mauldon J, Nuru-Jeter A (2016). Young women’s contraceptive decision making: do preferences for contraceptive attributes align with method choice?. Perspect Sex Reprod Health.

[22] Nilsson A, Ahlborg T, Bernhardsson S (2018). Use of non-medical contraceptive methods: a survey of women in western Sweden. Eur J Contracept Reprod Health Care.

[23] Statistics Sweden. https://www.scb.se/hitta-statistik/sverige-i-siffror/manniskorna-i-sverige/foraldrars-alder-i-sverige/#:~:text=Under%202021%20var%20medel%C3%A5ldern%20f%C3%B6r,och%2034%2C3%20f%C3%B6r%20papporna. Accessed 3 Feb 2023.

[24] Duane M, Contreras A, Jensen ET, White A (2016). The performance of fertility awareness-based method apps marketed to avoid pregnancy. JABFM.

[25] Holt K, Reed R, Crear-Perry J, Scott C, Wulf S, Dehlendorf C (2020). Beyond same-day long-acting reversible contraceptive access: a person-centered framework for advancing high-quality, equitable contraceptive care. Am J Obstet Gynecol.

[26] Iversen L, Sivasubramaniam S, Lee AJ, Fielding S, Hannaford PC (2017). Lifetime cancer risk and combined oral contraceptives: the Royal College of General Practitioners’ Oral Contraception Study. Am J Obstet Gynecol.

[27] Dehlendorf C, Krajewski C, Borrero S (2014). Contraceptive counseling: best practices to ensure quality communication and enable effective contraceptive use. Clin Obstet Gynecol.

[28] Schivone GB, Glish LL (2017). Contraceptive counseling for continuation and satisfaction. Curr Opin Obstet Gynecol.

[29] Stern J, Molin MS, Fernaeus M, Georgsson S, Carlsson T (2022). Contraceptive counseling about adverse reactions of intrauterine contraception: exploration of narratives found in web-based discussion boards. Midwifery.

[30] The Swedish Internet Foundation. 2018. https://internetstiftelsen.se/docs/Svenskarna_och_internet_2018.pdf. Accessed 230203.

